# Editorial: Circadian Control of Immunity 

**DOI:** 10.3389/fimmu.2020.618843

**Published:** 2020-11-30

**Authors:** Koichi Ikuta, Christoph Scheiermann

**Affiliations:** ^1^ Laboratory of Immune Regulation, Institute for Frontier Life and Medical Sciences, Kyoto University, Kyoto, Japan; ^2^ Department of Pathology and Immunology, Faculty of Medicine, University of Geneva, Geneva, Switzerland; ^3^ Biomedical Center, Faculty of Medicine, Institute of Cardiovascular Physiology and Pathophysiology, Ludwig Maximilians University, Planegg-Martinsried, Germany; ^4^ Walter-Brendel-Centre of Experimental Medicine, University Hospital, Ludwig Maximilians University, Munich, Germany

**Keywords:** circadian immunity, innate immunity, adaptive immunity, leukocyte migration, control of circadian immunity

## Introduction

The circadian clock influences virtually every aspect of life in mammals. Circadian clocks allow the organism to adjust to and anticipate recurring changes in their rhythmic environment, allowing for a better fitness and survival (1, 2). Within the immune system, it has long been known that the organism’s response to immune stimulation is highly time-of-day dependent, resulting in over-activation and even death (3). Thus, while the existence of an overall oscillation in the response to an immune stimulant over 24 h has been recognized decades ago, the molecular mechanisms behind these features remained elusive until recently. In this special issue, we highlight the recent developments in the fast-growing field of circadian immunology.

Historically, the influence of the circadian clock on the innate system has been recognized first and thus this is the aspect of circadian immunology that we know most about so far. This is also due to the fact that time-of-day was thought to exert its influence most strongly on the acute effect of the immune system. More recent data, however, demonstrate that also the adaptive immune system is clock-controlled, although also here previous observations had already indicated an impact of time-of-day (4). This review series focuses on the recent developments in the circadian aspects of immune cell functions, providing an overview over the innate and adaptive immune system, neuronal and hormonal control as well as the influence of the microbiome on rhythmic immunity.

One of the most prominent features of a rhythmic immune system is the rhythmic fluctuation of immune cells in blood. Recent data indicate that this reflects their redistribution from blood to tissues, which is reviewed by Yuan et al. Thus, the temporal difference in the presence of certain immune cells at specific sites in blood and tissues will certainly contribute to any differences in the immune response.

## Rhythms in the Innate and Adaptive Immune System

The cell type that so far has been investigated in most detail is the macrophage (5, 6). Timmons et al. provide an in-depth overview over the recent developments with respect to their time-of-day dependency. Neutrophils, the most abundant immune cells in human blood, have remained a much less-studied leukocyte subset but have also been shown to be highly rhythmic in their activity and their trafficking patterns (7, 8). Aroca-Crevillén et al. provide an overview into the recent scientific developments in this aspect. Pourcet and Duez give insights into the rhythmic activation of the inflammasome, a key inflammatory signalling complex that integrates inflammatory input with immune cell output (9). In addition, the adaptive immune system is highly rhythmic and this has been demonstrated in detail with respect to allergic reactions. Nakao sums up the recent data into the role of the circadian clock in allergy.

## Neural and Hormonal Control

An important question that is currently an active field of research is how immune cells and immune responses in general are entrained. Recent data indicate that both glucocorticoids (10) as well as the sympathetic nervous system (11, 12) can govern these oscillations. Leach and Suzuki as well as Shimba and Ikuta discuss these recent developments with respect to adrenergic nerves as well as glucocorticoids, respectively. Interestingly, these oscillations are not only affecting mature leukocyte populations but are also observed at the level of hematopoietic stem and progenitor cells (13). García-García and Mendez-Ferrer discuss the recent development in the field with respect to immature hematopoietic cell populations.

## Microbiota

While the field is currently trying to better understand how a complex, multicellular organism orchestrates rhythmic immune reactions, the level of complexity is increased even further by the role that exogenous factors, predominantly the commensal microbiota, play in this. Aspects of the gut microbiota have been shown to be strongly rhythmic (14) and Butler and Gibbs, as well as Kubo sum up the recent insight into this role.

## Conclusions

The circadian control of immunity is achieved in a cell autonomous manner by clock genes and can be entrained with the help of adrenergic nerves and glucocorticoids. This collection of review articles on the Research Topic “Circadian Control of Immunity” provides the latest and comprehensive update in this rapidly growing field of immunology research.

## Author Contributions

Both authors contributed equally to the manuscript. All authors contributed to the article and approved the submitted version.

## Funding

Work in KI’s lab is supported by the Japan Society for the Promotion of Science (JSPS) KAKENHI Grant Numbers 20K21525 and 18K15184. Work in CS’s lab is supported by the Swiss National Science Foundation (SNF, 310030_182417).

## Conflict of Interest

The authors declare that the research was conducted in the absence of any commercial or financial relationships that could be construed as a potential conflict of interest.
